# Assessing bone thickness in the infrazygomatic crest area aiming the orthodontic miniplates positioning: a tomographic study

**DOI:** 10.1590/2177-6709.22.4.070-076.oar

**Published:** 2017

**Authors:** Aline Rode Santos, Marcelo Castellucci, Iêda Margarida Crusoé-Rebello, Márcio Costa Sobral

**Affiliations:** 1 Universidade Federal da Bahia, Departamento de Ortodontia (Salvador/BA, Brasil).; 2 Universidade Federal da Bahia, Departamento de Radiologia Bucal (Salvador/BA, Brasil).

**Keywords:** Measurements, Tomography, Orthodontics.

## Abstract

**Introduction:**

Due to the increasing use of miniplates for anchorage purposes in orthodontics, it is very important to know more about infrazigomatic crest anatomy (thickness), in adult patients.

**Objectives:**

Evaluate the infrazygomatic crest region thickness, in adult (male and female) patients.

**Methods:**

Cone-beam computerized tomography (CBCT) images from 40 patients were used to assess cross-sectional measurements of the infrazygomatic crest region. Measurement 1 considered thickness 2 mm above the distobuccal root of the permanent maxillary first molar, while measurement 2 was taken 2 mm above the first measurement.

**Results:**

The mean thickness of the infrazygomatic crest in males was 3.55 mm for measurement 1 and 2.84 mm for measurement 2, while in females these were 2.37 mm and 2.24 mm, respectively.

**Conclusion:**

The authors concluded that the overall mean thickness of the infrazygomatic crest was 2.49 mm with respect to measurement 1, and 2.29 mm for measurement 2, with no statistically significant differences between gender.

## RESUMO

**Introdução:**

devido ao aumento do uso de miniplacas para ancoragem em Ortodontia, torna-se de fundamental importância compreender melhor a anatomia da crista infrazigomática (espessura) em pacientes adultos.

**Objetivos:**

avaliar a espessura da crista infrazigomática em pacientes adultos (sexos feminino e masculino).

**Métodos:**

foram utilizadas tomografias computadorizadas de feixe cônico (TCFC) de 40 pacientes, para avaliar medidas da região da crista infrazigomática, no sentido transversal. A Medida 1 verificou a espessura mm acima da raiz distovestibular do primeiro molar permanente superior, enquanto a Medida 2 foi realizada 2 mm acima da primeira medida.

**Resultados:**

a espessura média da crista infrazigomática encontrada no sexo masculino foi de 3,55 mm para a Medida 1 e de 2,84 mm para a Medida 2, e no sexo feminino foi de 2,37 mm e de 2,24 mm, respectivamente.

**Conclusão:**

os autores puderam constatar que a espessura média da crista infrazigomática foi de 2,49 mm para a Medida 1, e de 2,29 mm para a Medida 2, sem diferença estatisticamente significativa entre os sexos.

## INTRODUCTION

One of the most frequently problems in orthodontics is to achieve the anchorage necessary to obtain a desired tooth movement.^1^ ^-^ ^4^ Conventional approaches employ the anchorage potential of existing teeth when a large number of these can resist the movement of a small number. This usually requires the use of auxiliary devices, such as intermaxillary elastics and/or headgear, but a negative aspect is that these devices depend on patient’s cooperation.^1^ ^,^ ^2^ ^,^ ^3^ ^,^ ^5^ ^-^ ^8^ The need to eliminate undesirable effects and, at the same time, maximize anchorage, has led to the development of skeletal anchorage systems utilizing osseointegrated implants, mini-implants and miniplates. These devices do not allow for the movement of the anchorage unit during orthodontic mechanics^9^ and they can be used 24 hours a day, offering an alternative method that better controls side effects.^8^

Titanium miniplates are temporary anchorage devices that not only provide better stability than mini-implants, but also are more resistant to stronger forces.^7^ ^,^ ^10^ They are placed at a greater distance from the root apexes, allowing distal movement around the arch as there is no interference between the fixed device and dental roots.^7^ ^,^ ^8^ ^,^ ^11^ These miniplates can be used for multiple purposes, such as direct or indirect anchorage for different types of tooth movement,^12^ in addition to providing skeletal anchorage for maxillary protraction.^13^ However, they require surgical procedures for placement and removal, which must be performed by a qualified surgeon due to increased complexity at this anatomical site.^7^ ^,^ ^10^

According to De Clerck et al,^12^ due to the location and solid bone structure, the inferior border of the maxillary zygomatic buttress, also known as the infrazygomatic crest, located between the first and second molars, is the chosen site for the placement of miniplates with the purpose of using the skeletal anchorage system, placing the miniplates at a safe distance from the roots of the maxillary molars.

Anatomically, the infrazygomatic crest has two cortical plates, a vestibular one and the lateral wall of the maxillary sinus. This anatomical advantage allows bicortical fixation and it contributes to improved primary stability of the screw.^14^ However, the infrazygomatic crest area is 2-5 mm thick, while miniscrews are approximately 5-7 mm long, which may cause perforation of the maxillary sinus during its placement.^8^

Due to the frequent use of skeletal anchorage, it is extremely important to conduct studies to assess the thickness of the infrazygomatic crest to better understand its anatomical dimensions, providing safer surgical procedures and minimizing possible failures.

The aim of this study was to verify the thickness of the infrazygomatic crest and compare it between male and female adult subjects, by using coronal slices from cone-beam computerized tomography (CBCT) imaging.

## MATERIAL AND METHODS

The present study employed CBCT images of 40 patients from a post-graduate course in orthodontics. Of the included patients, 18 were male (45%), 22 female (55%), aged 22-56 years (mean age of 31 years), and all full-filled the following criteria: need for maxillary bone anchorage during orthodontic treatment, presence of the permanent maxillary first molars; over 21 years of age; no presence of bone lesions in the maxillary region.

In order to test the sample power, it was performed a power analysis using R-software (www.r-project.org, version 3.3.2). It was found a power of 80%, based on the significance level of alpha of 0.01 and the effect size of 0.85.

This project was approved by a Institutional Ethic’s Committee, protocol number 905.596. All included patients were required to sign a informed consent form, allowing their exams to be used for research purposes.

The study assessed the thickness of the infrazygomatic crest by obtaining cross-sectional measurements using coronal slices from cone-beam computerized tomography (CBCT) images. CBCT images were obtained using an i-CAT^®^ device (Imaging Sciences International, Hatfield, PA, USA) with an acquisition protocol of 120 Kvp, 47 mA, 0.4 mm-thick slices, 0.4-mm voxel size, 20 x 25 cm field of view (FOV) and an acquisition time of 40 seconds. CT scans were performed with patients seated so that the Frankfort horizontal plane was parallel to the ground, in a maximum intercuspation position.

The Digital Imaging and Communications in Medicine (DICOM) file format was used to compose three-dimensional reconstructions of each patient’s facial structure using Dolphin Imaging^®^ software, version 11.5 Premium (Dolphin Imaging & Management Solutions, Chatsworth, USA). After the image processing, the head orientation in each digital image was standardized according to the sagittal, coronal and axial planes. In the frontal view, the patient’s median line was aligned in accordance with the orientation line of the software and the right and left frontozygomatic sutures were marked. In the lateral view, the right orbital and right porion points were located and positioned to coincide with the Frankfort horizontal plane.^15^

**Figure 1:**
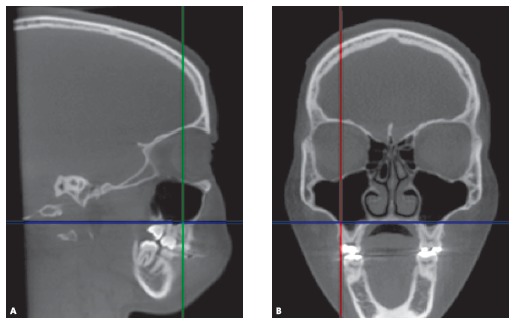
Sagittal slice (A) used to obtain coronal slice (B), consisting of the apex of the distobuccal root of the permanent first maxillary molar.

Next, the apex of the distobuccal root of each permanent first maxillary molar was located using a sagittal CT slice to obtain a coronal view on each side (Fig 1).

**Figure 2:**
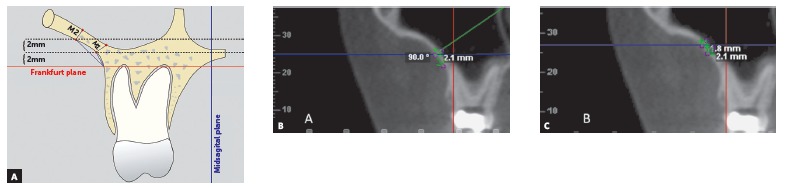
Measurement of infrazygomatic crest thickness, perpendicular to the buccal surface (A). Illustrative picture at 2 mm (B) and at 4 mm (C) above the apex of the distobuccal root of the permanent first maxillary molar.

Two measurements were obtained along the infrazygomatic crest with the aid of the Digitize-measurement tool in the Dolphin Imaging program. The first measurement (measurement 1) was performed at 2 mm above the distobuccal root apex of the permanent first maxillary molar along the buccal wall of the infrazygomatic crest, specifically where the horizontal axis of the program coincided with the oral surface of the infrazygomatic crest. The next measurement (measurement 2) was performed 2 mm above the first one, maintaining the same procedure used in measurement 1 (Fig 2).

### Statistical analysis

Prior to taking measurements, in order to calibrate the examiner, 10 scans were selected. All digital measurements were performed by a single previously calibrated operator under identical conditions at two different times, with a two-week interval. To verify agreement among the measurements, Lin’s concordance test was used, obtaining a result of 0.98, considered an almost perfect concordance.

A database was created in Excel 2003 and analyzed in the R software version 3.1.0 (R Foundation for statistical computing, Wien, Österreich). A descriptive analysis (absolute/relative frequency, mean, standard deviation and median) was performed to identify the general and specific characteristics of the study sample. The normality assumptions were verified using Kolmogorov-Smirnov and Shapiro-Wilk tests and the Levene’s test of variance homogeneity. With respect to the data presenting normal distribution, parametric testing was employed, while in the non-normal distributions, non-parametric testing was used.

With the objective of comparing the median thickness of the measured crest with the hypothesized measurement of a standard 5-mm long miniscrew, one-sample Wilcoxon test was used. Student’s *t*-test was used to verify significant differences among the different heights. Mann-Whitney test was used to verify if there were any significant differences in measurements according to gender. Wilcoxon test for paired samples was used to compare the measurements between the two sides.

The significance level for this study was 5%. The results are presented in comparative tables.

## RESULTS

The present study found that the overall mean thickness of the infrazygomatic crest was 2.49 mm with respect to measurement 1, and 2.29 mm for measurement 2. Table 1 shows that no statistically significant differences were observed when comparing genders.

**Table 1:** Comparison of thickness of the infrazygomatic crest between sexes (Mann-Whitney test).

VARIABLES	sex						p-value
	MALE			FEMALE			
	Mean	Standard deviation	Median	Mean	Standard deviation	Median	
Measurement 1	2.62	1.41	2.2	2.37	1.0	2.2	0.747
Measurement 2	2.34	1.13	1.85	2.24	0.73	2.1	0.692

Table 2 delineates the statistically significant differences seen between measurements 1 and 2 (*p* = 0.019).

**Table 2:** Comparison between measurements 1 and 2 (Student’s t-test).

Measurement 1			Measurement 2			p-value
Mean	Standard deviation	Median	Mean	Standard deviation	Median	
2.49	1.21	2.2	2.28	0.94	2.0	0.019

Table 3 indicates that statistically significant differences were found between the right and left sides with respect to measurement 2 (*p* = 0.002).

**Table 3:** Comparison between the right and left sides (Wilcoxon test).

VARIABLES	Right side			Left side			p-value
	Mean	Standard deviation	Median	Mean	Standard deviation	Median	
Measurement 1	2.24	0.9	2.0	2.74	1.42	2.4	0.111
Measurement 2	2.0	0.71	1.9	2.52	1.08	2.25	0.002

## DISCUSSION

The Schneiderian membrane, which is attached to the bordering bone of the maxillary sinus, is characterized by a periosteum overlaid with a thin layer of pseudociliated stratified respiratory epithelium, constituting an important barrier for the protection and defense of the sinus cavity. Its integrity is essential to normal sinus function.^16^

According to Reiser et al,^17^ when implants extend less than 2 mm into the maxillary sinus, the sinus membrane becomes elevated, which favors healing as it allows for the formation of a blood clot that provides a scaffold for bone formation in this region. When the implant extends further into the maxillary sinus, i.e. greater than 2 mm, the Schneiderian membrane becomes perforated, which may result in the discharge of bone fragments inside the maxillary sinus, thus compromising healing ability, increasing the occurrence of sinusitis.

In a clinical and experimental study of the effects caused by the penetration of osseointegrated dental implants into the nasal cavity and the maxillary sinus, Brånemark et al^18^ noted that titanium implants did not cause side effects and were well-anchored in bone. The authors believe that no side effects were seen because these implants became osseointegrated into the bone structure, which promoted direct contact between the implant, bone and soft tissue. This direct connection between the implant and the hard and soft tissues creates a barrier against the migration of microorganisms, and inhibits the inflammatory process around the implant. In essence, osseointegration protects the implant. On the other hand, according Adell et al,^19^ when osseointegration does not occur, a fibrous tissue covers the implant, normally leading to the induction of an inflammatory process followed by bone resorption and implant loss.

In order for implants to be placed into a suitable bone surface, the anatomy of the infrazygomatic crest must be known by means of specific exams, such as CBCT.

The present study demonstrates that the infrazygomatic crest is significantly thinner than the length of the miniscrews commonly used in this region, as the mean thickness of the crest was found to be 2.49 mm for measurement 1, and 2.29 mm for measurement 2. The present findings corroborate those of Liou et al,^14^ who found a thickness of 2.9 ± 0.9 mm in the lateral wall of the maxillary sinus (where miniplates were placed) and Baumgaertel and Hans,^20^ who found a mean thickness of 3.87 mm at 2 mm from the apex of the distobuccal root of the first molar and 2.98 mm at 4 mm from the apex of the distobuccal root.

Liou et al^14^ and Lee et al^21^ found no statistically significant differences between the measurements on the right and left sides. However, the present results show a statistically significant difference between the right and left sides with respect to measurement 2, among female patients - although this data is not clinically relevant, since the differences found were very small in comparison to the 5-mm miniscrew size.

Lee et al^21^ found that the infrazygomatic crest was clinically thicker in male patients than in female patients. In the present study, no statistically significant differences were detected between sexes. In fact, marked individual variations in these measurements were observed irrespective of sex, which is in agreement with the findings of Farnsworth et al.^22^

The present study found that the average thickness of the infrazygomatic crest was smaller when measured further from the root apex, corroborating the results reported by Baungaertel and Hans,^20^ who found greater risk of maxillary sinus perforation when miniscrews were placed in a more cranial orientation.

Baungaertel and Hans^20^ stated that great individual variation exists in the thickness of the infrazygomatic crest. Indeed, the present study also found measurements ranging from 0.9 to 7.4 mm, which is probably due to differing root lengths, maxillary sinus pneumatization, buccolingual inclination of the maxillary first molar, and the height of the alveolar processes among the individuals studied, all of which are determinants to the available bone depth for miniscrew placement. However, according to Kravistz and Kusnoto,^23^ if the maxillary sinus membrane is perforated during miniscrew placement, immediate removal must not occur due to its small diameter. Orthodontic therapy must continue and the patient should be followed to avoid the possible development of sinusitis and mucocele.

Kim et al^24^ conducted a computed tomographic study to observe the placement of 31 miniplates, placed between the roots of the posterior teeth of 18 patients. To conduct the study, 74 screws, 4-mm long and 1.5 mm in diameter, were used. Their results showed that, of 74 miniscrews, 39 perforated the maxillary sinus, with mean exposure of 1.31 ± 0.72 mm. Among these, only 3 miniscrews protruded more than 2 mm into the maxillary sinus (2.37, 2.95, and 3.41 millimeters). No miniscrews presented mobility or caused any further complications, such as sinusitis, swelling or peri-implant inflammation. However, to conduct the study, Kim et al.^24^ only selected patients who had presented clinical stability for a period of six months after miniplate placement and, as such, their results do not confirm the absence of risk factors for maxillary sinus perforation. It is noteworthy that, in the present study, the thickness of the infrazygomatic crest was compared with the length of a 5-mm miniscrew. Studies performed by De Cleck et al,^12^ an internationally renowned author for his studies of miniplates for orthodontic anchorage, have indicated that this region is the best option for the placement of miniplates. 

Miyawaki et al^25^ observed in their studies that the stability of the miniscrew is not related to its length, but rather to its diameter, as 1-mm thick miniscrews demonstrated less stability than 1.5 and 2.3-mm miniscrews. Kim et al^2^ also agree that the interface between the miniscrew and cortical bone is an important factor affecting the stability of the miniscrew. Myawaki et al^25^ further suggest that the thickness of the cortical bone should be verified by CT scan prior to the placement of these anchoring devices, which could indicate the use of miniscrews with a diameter greater than 2.3 mm in the case of thin cortical bone, thereby providing greater stability by increasing the contact between the cortical bone and the miniscrew.

## CONCLUSION

The authors concluded that the overall mean thickness of the infrazygomatic crest was 2.49 mm with respect to measurement 1, and 2.29 mm for measurement 2, with no statistically significant differences between sexes.

## CLINICAL CONSIDERATIONS

The present study found the mean thickness of the infrazygomatic crest to be significantly thinner than the length of miniscrews commonly used in this region, which may cause maxillary sinus perforation if miniscrews of 5 mm or longer are used for miniplate anchorage. As a result, it is recommended to manufacture shorter screws, which would enable dental surgeons to offer safer procedures to their patients. However, the literature is controversial regarding the side effects of perforation and therefore, further study is necessary. Nonetheless, the risk of miniplate instability due to insufficient bone thickness, in addition to the development of sinusitis or other inflammatory processes, must be taken into account by orthodontists, and patients must be informed of the relevant risks posed by these types of procedures.
